# If you build it, they will come: unintended future uses of organised health data collections

**DOI:** 10.1186/s12910-016-0137-x

**Published:** 2016-09-06

**Authors:** Kieran C. O’Doherty, Emily Christofides, Jeffery Yen, Heidi Beate Bentzen, Wylie Burke, Nina Hallowell, Barbara A. Koenig, Donald J. Willison

**Affiliations:** 1 Department of Psychology, University of Guelph, Guelph, ON N1G 2W1 Canada; 2 Centre for Medical Ethics, Faculty of Medicine, University of Oslo, Oslo, Norway; 3 Norwegian Research Center for Computers and Law, Faculty of Law, University of Oslo, Oslo, Norway; 4 Norwegian Cancer Genomics Consortium, Oslo, Norway; 5 Department of Bioethics & Humanities, University of Washington, Seattle, USA; 6 Ethox Centre, Nuffield Department of Population Health, University of Oxford, Oxford, UK; 7 UCSF Bioethics, Institute for Health & Aging, University of California, San Francisco, USA; 8 Institute of Health Policy Management and Evaluation | Joint Centre for Bioethics, University of Toronto, Toronto, Canada; 9 Department of Clinical Epidemiology and Biostatistics, Faculty of Health Sciences, McMaster University, Hamilton, Canada

**Keywords:** Health data, Privacy, Secondary uses, Discrimination, Health research, Biobanks, Data sharing, DNA

## Abstract

**Background:**

Health research increasingly relies on organized collections of health data and biological samples. There are many types of sample and data collections that are used for health research, though these are collected for many purposes, not all of which are health-related. These collections exist under different jurisdictional and regulatory arrangements and include:

1. Population biobanks, cohort studies, and genome databases
2. Clinical and public health data
3. Direct-to-consumer genetic testing
4. Social media
5. Fitness trackers, health apps, and biometric data sensors

Ethical, legal, and social challenges of such collections are well recognized, but there has been limited attention to the broader societal implications of the existence of these collections.

**Discussion:**

Although health research conducted using these collections is broadly recognized as beneficent, secondary uses of these data and samples may be controversial. We examine both documented and hypothetical scenarios of secondary uses of health data and samples. In particular, we focus on the use of health data for purposes of:

- Forensic investigations
- Civil lawsuits
- Identification of victims of mass casualty events
- Denial of entry for border security and immigration
- Making health resource rationing decisions
- Facilitating human rights abuses in autocratic regimes

**Conclusions:**

Current safeguards relating to the use of health data and samples include research ethics oversight and privacy laws. These safeguards have a strong focus on informed consent and anonymization, which are aimed at the protection of the individual research subject. They are not intended to address broader societal implications of health data and sample collections. As such, existing arrangements are insufficient to protect against subversion of health databases for non-sanctioned secondary uses, or to provide guidance for reasonable but controversial secondary uses. We are concerned that existing debate in the scholarly literature and beyond has not sufficiently recognized the secondary data uses we outline in this paper. Our main purpose, therefore, is to raise awareness of the potential for unforeseen and unintended consequences, in particular negative consequences, of the increased availability and development of health data collections for research, by providing a comprehensive review of documented and hypothetical non-health research uses of such data.

## Background

Organised collections of biological samples and personal information have become increasingly important for health research, particularly in achieving translational aims to improve primary prevention, diagnosis, and treatment [1]. Increasing reliance on biobanks and health databases has led to significant escalations in funding to support the collection of tissue and information as is evident, for example, in the recently announced U.S. Precision Medicine Initiative [2, 3]. That the use of health data for research is associated with significant ethical challenges is well recognised. A vibrant community of ELSI (ethical, legal, social implications) scholars has been debating issues such as informed consent requirements for biobanks, privacy implications of long-term storage of health data and data sharing, and the return of findings to research participants [4–11]. Most of these debates occur in the context of research ethics frameworks aimed at the protection of individual research participants. However, information collected in health research, particularly genomic data, also has implications for social groups (family and identifiable ethnic groups) to which individuals belong, and for society as a whole.

Although there are many effective mechanisms in place to protect personal data collections, there has been relatively little consideration of the potential consequences of the proliferation of organised collections of detailed and highly personal information on a broader societal level. In particular, there is a paucity of attention to non-research uses for which such data collections might be employed, irrespective of the original intentions of the builders and the expressed purposes of the platforms. New data and new forms of research, and most importantly, new research platforms and institutions, create new possibilities; they therefore establish new societal affordances beyond their utility for health research.

The purpose of this paper is to present a comprehensive review of potential uses of health data collections that are largely unforeseen and unintended by the biomedical research community. In so doing, we acknowledge the socially valuable health research that has been conducted through secondary use of data. We also acknowledge the important role the ELSI community has played in facilitating ethical use of such data. Nevertheless, we believe that key issues have not received sufficient attention, which becomes apparent when one considers some of the implications of living in a society in which the collection and electronic storage of health data have become increasingly normalized.

We begin by outlining the types of health data collections we are concerned with. These include collections established specifically for health research, as well as some collected for other reasons, but which have subsequently become valuable for health related research. In particular, we focus on human tissue biobanks, public health data collections, and data accumulated by private entities, such as direct-to-consumer genetic testing companies, Facebook, and fitness trackers. We then consider potential secondary uses of these data collections that are outside of, or even contrary to, the scope and purpose of the types of collections we have outlined. In our analysis, we consider secondary uses of health data for which instances have already been documented internationally (such as forensic investigations, civil law suits, and mass casualty events). We then consider a hypothetical scenario, the potential for health databases to be used for health care rationing decisions, to illustrate how shifting social priorities and interests might lead to repurposing of health data collections. Finally, we consider some historical examples of how population data collections subsequently came to facilitate large scale human rights abuses.

Taken together, these examples illustrate that ethical analysis of the development and use of health data collections is inadequate if it is limited to narrow consideration of risks and benefits, especially when this is focused on individual research participants. Rather, the development of health data collections and the potential uses they enable constitute critical shifts in the societal environment that should be subject to democratic scrutiny and decision-making procedures. Research ethics oversight (in the form of Institutional Review Boards and similar bodies) is not capable of guarding against the type of uses we discuss. Our intention is to raise awareness of these issues in the hope that they will be discussed more prominently in ELSI and related communities and, ultimately, lead to robust protections.

## Discussion

### Organised collections of personal health data

Data that are useful for health research come from a range of sources and can be made available to researchers through a variety of mechanisms. We are concerned here primarily with data that are stored for long or indefinite periods of time as part of an organised collection. In particular, we focus on population biobanks, cohort studies, and genome databases; clinical and public health records; genetic data collected through direct-to-consumer genetic testing; data collected through social media; and data collected by fitness trackers, health apps, and biometric data sensors. In this context, we are cognisant of important differences in legal definitions and other uses between biological data, information, databases, and biological samples [12–14]. For the purposes of this paper, we are concerned with the societal consequences of collections including all of these categories.

### Population biobanks, cohort studies, and genome databases

Researchers increasingly access data from central repositories and are expected to contribute data they generate to such repositories [15–17] (see also the United States NIH policy on data sharing) [18]. Driven largely by the rationale of maximizing the utility of data generated by public funding for health research [19], many journals now require that researchers make their data publicly available as a condition of publication (e.g., PLOS, Nature) [20, 21]. Similarly, population biobanks collect and store biological samples and associated personal health and demographic information for the purpose of facilitating health research. Many population biobanks are also associated with ongoing follow-up of participants and so are best characterized as cohort studies (e.g., The Canadian Longitudinal Study on Aging) [22]. In contrast to data gathered for the purpose of a particular study, data gathered for population biobanks are intended, from the outset, to be of benefit to as many research studies as possible. Such biobanks thus rely on significant public investment to create a research infrastructure that is widely accessible for an indefinite period of time.

The types of data that are collected and shared vary across studies and biobanks, but may include: demographic information, disease history, health related behaviours, psychosocial information, a wide variety of biological measures, brain scans, and genetic information [23, 24]. Depending on the type of collection, directly identifying information may be linked to individual level records [25]. With scientific and technological advances, new forms of data collection are being enabled. For example, there is now a large number of whole genome and exome sequences that have been generated as part of different research endeavours, and efforts are under way for creating data storage mechanisms that would increase accessibility of these sequences to scientists [26]. There is also an increasing use of natural language processing in mining of clinical data [27, 28], which makes strategies of anonymization difficult to implement.

In the past, research ethics protocols often assumed a direct relationship between research participants and the researcher(s) who recruited them for a particular study, and this expectation is still evident among many research participants [29]. When data are shared beyond the original researchers or when data are collected for large research platforms, which will be used by multiple researchers, stewardship of data often cannot be located meaningfully with a particular researcher. Personal data collections are therefore associated with institutions (i.e., the biobank, or the health data repository), rather than individuals. While publics and research participants, in particular, tend to be supportive of having their samples used in future research, this view depends on a number of factors including: the country where the research is conducted, characteristics of the research participants, the type of sample, and who will have access to the samples [30, 31]. The importance of appropriate governance for biobanks and data collections has been widely acknowledged [32–34], though in practice, governance of these resources tends to be located within the scientific community. For example, governance of the NIH Database of Genotypes and Phenotypes (dbGaP) and other U.S. NIH repositories is controlled by data access committees appointed by NIH, which are limited to federal employees and generally consist of scientists working within NIH [35]. dbGaP specifically prohibits institutions submitting data from adding their own safeguards over and above enforcement of consent (e.g., in the process of submitting data to dbGaP research institutions would not be allowed to add institutional requirements to safeguard samples/data, beyond enforcement of consent).

### Clinical and public health data

Every interaction between an individual and a health care provider leads to information being recorded about symptoms, health histories, and past or current medications. This information is, in the first instance, directly linked with the patient’s name and other personal identifiers (e.g., address, health insurance number, etc.). Much of this information is also shared with pharmacies (to fill a prescription), health insurance companies (for reimbursement), labs (where testing may be completed) and other test locations. The primary purpose of collecting this information is to provide optimal health care for individuals and communities.

What then happens with this information varies by jurisdiction. In Canada, for example, such data are sent to the provincial health insurer, in part to allow physicians and other health care specialists to be paid. If an individual is hospitalized, further data are collected and analyzed to maintain records of: wait times for care, type of care provided, and ultimate resolution (for example, discharge to long-term care). This information is also collated centrally by the Canadian Institute for Health Information (CIHI) for research on quality of care. In the US, although health care systems vary by state and health data are not automatically pooled, Medicare data are aggregated and studied, much like the Canadian data. Also, specific data collation efforts collect various types of health data from health care organizations (e.g., the U.S. CDC’s collection of ambulatory health data) [36], and information is also collated by insurance companies to determine eligibility and pay the providers.

Regardless of the type of health care system, individual health information may be shared to some degree with other health care providers for the purposes of clinical care [37]. In addition, some of these data may be shared outside of the health care system for the purposes of research. Across jurisdictions there is also a trend to move increasingly towards electronic medical records (EMR), which facilitate data sharing for improving clinical care and research [38]. In some instances, there has been significant controversy surrounding the use of health data collected for clinical purposes, for subsequent research purposes. For example, in Denmark, over a period of several years the Danish General Practice Database reused confidential information about patients’ diagnoses for purposes of analysis and research without patients’ or physicians’ consent and awareness [39].

In addition to health information collected at point of care, information and biological samples are also collected in public health and screening programs. For example, many countries have newborn screening (NBS) programs. The primary purpose of these programs is to test for rare diseases such as phenylketonuria that are critical to detect in the early days of life [40]. A blood sample is collected from infants to conduct these diagnostic tests, but once the sample is collected, there is ethical ambiguity about what can or should be done with it [41], [42]. Often these collections are not “consented” and how they are governed and whether samples may be used for other purposes varies dramatically across jurisdictions [43, 44]. There has been significant public controversy about secondary research uses of newborn bloodspots [45] and while research shows that in the US the public tends to be supportive of the program, there is a preference for parental opt-in for secondary research uses [46].

For different types of health data and across jurisdictions, there is variation as to whether individuals are informed about the fact that their personal information is collected and used for research. If individuals are informed, there is variation in whether consent processes are in place, and whether these are opt-in or opt-out based (in many cases legislation is in place that allows for research use of samples and data without consent for quality assurance purposes). This strong focus on collecting, storing, and sharing personal and aggregate health data is frequently rationalized as supporting the development of a more “personalized medicine” [47] or “precision medicine” [48].

### Direct-to-consumer genetic testing

Since their development, genetic tests have been offered to individuals through referral within the health care system in most jurisdictions in which they are available. In recent years there has been a proliferation of commercial companies that offer genetic tests for purchase “direct-to-consumer” (DTC) [49]. These genetic tests claim to provide such information as how individuals will respond to certain drugs, verification of paternity, tracing of ancestry, and identifying propensities for psychological or physical health conditions [50–53]. In procuring such services, an individual submits a DNA sample, often with additional personal information.

In the process of providing such services, DTC genetic testing companies accumulate large amounts of personal information on individuals. However, it is not clear how such data are managed. In 2012, 86 companies offered genetic testing services online [54]. Of these, only 28 disclosed what would be done with samples and information after the commercial transaction was concluded. It has become clear that for at least some of these companies, the financial gain from the transaction with individuals procuring the genetic test is not intended to be the primary source of revenue. Rather, the business model relies on the sale of the data to third parties [55, 56]. Interestingly, private firms operating EMRs (see previous section) also adopt business models that assume that a substantial portion of their revenue comes from analysis of data in the records they keep for employers and insurers for private insurance plans [57].

### Social media

In contrast to the types of health data collections considered above, social media illustrate an accumulation of personal information that is more complex and sensitive to individual disclosure decisions. The information inherent in data from social media is also more dynamic and potentially far richer than other data sources in that it includes historical and up-to-date information about relationships, associations, and behaviours.

Social media are receiving increasing attention from social scientists, but are rarely considered in the context of health information. Many individuals willingly reveal very large amounts of personal information on social media sites, which enables the construction of an extensive picture of their health, lifestyle, biography, and behavior. For example, on sites such as Facebook, it is quite common for individuals to share personally identifying information such as birthdays, email addresses, addresses, and current or previous schools [58]. People also commonly share behavioral and biographical information such as daily activities, interests, habits, hobbies, marital or relationship status, family and friend connections, all documented through comments, photos, and videos. Although people do not typically share their social security number (SSN), it has been shown that SSNs and other personal data can be inferred by aggregating information that people share online and publicly available information about them [59]. Other less commonly shared information such as sexual orientation can also be predicted from network connections [60]. Recent use of Facebook as a platform for research has resulted in widespread outrage, based primarily on the involvement of Facebook users in a study without their informed consent [61].

### Fitness trackers, health apps, and biometric data sensors

The widespread use of mobile and wearable devices such as smartphones, fitness trackers and their associated health applications (apps) allows the measurement of a vast range of activities and physiological characteristics for health and performance. From daily habits and activities that users enter, to data recorded by cell phones and other devices through GPS and accelerometer readings (e.g. step counts or distance travelled), mobile devices and apps can monitor people with little effort or awareness on their part. Private companies and health researchers alike are eager to exploit the rich, real-time data streams these generate. Apple, for example, recently released its ResearchKit [62] to facilitate the development of health research applications for their devices. The day ResearchKit was launched, apps were also released to aid researchers in studying heart disease, Parkinson’s disease, breast cancer, diabetes and asthma [63].

Irrespective of concerns about their effectiveness [64], what is certain is that such apps and devices enable the generation of highly organised collections of very personal information. Few apps or devices are subject to government regulation despite the fact that they collect health information or offer health advice [65]. Even apps that have been accredited (e.g., by the NHS in the UK) have been shown to fall short of data protection standards [66]. Perhaps even more disconcerting is the automatic and unobtrusive collection of biometric measurements enabled by wearable sensors. For example, in some computer games physiological measures are used to assess the arousal level of gamers with the goal of optimising game play difficulty on an individual basis [67]. Gamification has also been suggested as a way of increasing adherence to health app use [68]. However, increased use of wearable technology and other connected devices is not without risks and these devices have become available before the societal implications of the data they provide have been fully considered [69].

In summary, multiple mechanisms can be identified whereby personal health related data are collected and made available to researchers in an organized fashion. Arguably, none of these data are collected for nefarious purposes. Indeed, if even a small proportion of the promised benefits of “big data” are realized, there is likely to be much benefit in them. These collections also exist under different governance regimes: ethics of clinical care, public health ethics, law, research ethics protocols, and commercial contracts between individuals and service providers. The collections rely on different types of funding: public/governmental research funding, health care system funding, consumer funding, advertising revenue models, and mixed funding models. The various collections also have different intended time horizons.

### Unintended and unforeseen uses of health data collections

The possibilities inherent in analysis of “big data” have been heralded as beneficial for health and as potentially decreasing health care costs [70]. However, it is self-evident that data collected for one purpose might also be used for other purposes not intended or envisaged at the time of collection. Indeed, some of the examples outlined above, such as newborn blood spot collections and biorepositories in pathology laboratories, have already been repurposed for secondary uses (i.e., samples and data originally collected for clinical or public health purposes were subsequently used for research). It is, indeed, conceivable that data collected for research purposes will eventually be used for yet other purposes [71].

Graves [72] argues that electronic health records represent the kind of organized collection of data that is attractive to domestic governments for surveillance and protection of its own population, to hostile foreign governments for espionage and sabotage, to commercial entities for generating revenue, and to criminals for illegal forms of profit. Given this environment of potentially hostile interests in health data, Graves suggests a compelling metaphor:

*“Given our security design and likely attackers, our situation is something like this. It is as if we had each taken the valuable contents of our homes and deposited them, carefully indexed, in a huge warehouse somewhere in the country. We then give the warehouse owner and his employees careful instructions about who may access and use these valuables. Thoughtfully, the warehouse owner creates a special passage into the warehouse—a “portal”—by which we may access a few of our own possessions if we wish. The builder of the warehouse has equipped it with a very fancy lock (AES with 128- to 256-bit keys) that the owner may choose to use if he thinks he’s in a bad neighborhood. Meanwhile, there are four different large, well-trained, well-equipped, and materiel-hungry armies in easy marching distance. Will the warehouse owner and his employees lay down their lives, or will they open the lock? Or will they discover their warehouse is riddled with secret back doors, or that the armies have really big bolt cutters (secret supercomputers that can do a brute force attack on AES), or that the lock has an equally fancy secret master key?” (p. 113)*

Perhaps Graves’s metaphor is too dramatic. Moreover, some of the secondary, non-research, uses that might arise for health data collections may be deemed uncontroversial or even broadly beneficial. But it would be naïve to think that organized collections of health data are of no interest to those outside of the health care and research community. With this in mind, we now illustrate some brief examples of conceivable secondary uses of health data and biological samples.

### Forensic investigations

Health data and organised collections of DNA, in particular, have important forensic uses [73]. Samples and information collected for health purposes may include information about individuals that is not routinely available in data collections available to law enforcement (or other) agencies. Situations can therefore arise in which the investigative opportunities afforded by existing health databases are of great interest for forensic and related purposes. Many examples illustrate this point. In some cases, the urgency of the situations that prompted requests for database access has meant that decisions were made without sufficient opportunity to engage in a fuller consultative process or consideration of the range of potential privacy and ethical issues.

A high profile case in the United States implicated the Texas Department of State Health Services (DSHS) in providing 8350 de-identified NBS samples to researchers without parental knowledge or consent [74]. In the midst of a class action lawsuit brought against the DSHS in 2009, it was revealed that the DSHS also gave 800 samples to the Armed Forces DNA Identification Laboratory (AFDIL) to help create a national mitochondrial DNA database for use in forensic investigation of missing persons and cold cases [1, 75]. It is worth noting here that this was not just a one-off use of the NBS data, which might have been justified on particular grounds, but rather the development (and repurposing) of a DNA database for forensic purposes.

When the Swedish Minister for Foreign Affairs, Anna Lindh, was assassinated in 2003, police requested access to the suspected attacker’s DNA from a national NBS biobank to compare against DNA from the crime scene. Police were granted access, resulting in the conviction of the assassin [76]. It is important to note that in this case there was no court involvement. The biobank acceded to the police request directly [77]. In an armed robbery in Norway, a cancer patient was a prime suspect. The suspect had died six months after the robbery, but tissue samples had been collected in a hospital biobank. Police wanted to compare DNA found at the robbery site with the suspect’s DNA from the biobank sample. In this instance police access was denied by the Norwegian Supreme Court [78].

### Civil lawsuits

There are multiple cases involving claims of paternity, typically related to disputes over inheritance, that lead to requests or attempts to access DNA samples stored in biobanks. In another Norwegian case, a dispute relating to claims of biological kinship led to access being granted to tissue samples from a hospital biobank. In this case, the Norwegian Supreme Court argued that the claimant’s right to know his parents superceded laws prohibiting biobanks from giving access to samples without the sample donor’s consent, who in this case was dead [79].

### Mass casualty events

In a Swedish case, a temporary amendment to the law restricting access to biobanks was passed after the 2004 Asian tsunami to allow for the use of samples from national NBS registries for identification of victims [80]. The decision was based on arguments emphasizing the great benefit to family members of the victims, while assuming that there is no reason to believe that people whose samples are being used would object to this use (though it should be noted that this decision was not without its critics).

DNA analysis was also used for victim identification purposes following both the 9/11 World Trade Centre attack and Hurricane Katrina, although this was achieved through kinship analysis with DNA samples obtained voluntarily from family members [81]. However, following such cases, some bioethicists have argued in favor of third-party access to health databases and biobanks after mass casualty events [82].

### Border security and immigration

Contemporary examples of third-party access to health data or biobanks in the context of immigration and border security are comparatively rare. However, it can be argued that health databases afford similar opportunities to agencies wishing to exploit their potential. Following 9/11 many states have passed anti-terror legislation to intensify surveillance of “suspect populations” and control their movement [83]. Many of these laws, such as the *USA Patriot Act*, grant security agencies broad powers to access private records without notifying the subjects of those records that an invasion of their privacy is being contemplated. The *Patriot Act* in particular has implications for non-U.S. jurisdictions, like the government of British Columbia, that have contracted with U.S. corporations to manage their electronic health records. Such corporations would be compelled to grant access to health records as a result of an application by a security agency using a much less stringent standard of proof [84].

One case in which health data may have been accessed by border personnel involved a Canadian woman attempting to travel to the United States in 2013 and being denied entry on the basis of a medical history of depression and attempted suicide. The woman, author Ellen Richardson, has written openly about her struggles with depression and suicide, and reported to the Canadian Broadcasting Corporation (CBC) that border officials cited her hospitalization for depression in June 2012 as the reason for the denial of entry [85]. Further investigation by Canadian journalists did not clarify how U.S. border officials gained access to Richardson’s medical history, but revealed that contact with the police through 911 calls is routinely recorded in the database of the Canadian Police Information Centre. Sometimes these encounter reports include information about mental health. This database is shared with the U.S. Federal Bureau of Investigation and other U.S. security agencies [86]. Richardson, however, reported that her 2012 hospitalization only involved contact with medical personnel in an ambulance, and not the police. The possibility that U.S. border officials are accessing Canadian health information is being investigated by the Ontario Privacy Commissioner [85].

What these examples illustrate is that the mere existence of collections of health data and biological samples creates possibilities for action and use that would otherwise not have been available. In cases like the tsunami, redirecting use of these resources found strong social support in spite of some controversy. In contrast, the use of NBS samples in Texas for forensic purposes led to wide-spread public outcry. While recognising that there are legal barriers to law enforcement and/or governmental access to data and samples, these cases demonstrate the potential that exists for DNA originally obtained for the purposes of public health screening or health research to be repurposed for law enforcement. As Kaye [87] has argued, large, centrally managed health databases cannot but be of interest to law enforcement and forensic communities, who are themselves advocating for the creation of large databases or biobanks to aid investigations [88, 89]. These developments have led legal scholars in the U.S. to warn that assumptions about privacy and unreasonable search and seizure under the Fourth Amendment do not account for the expanded investigative and surveillance capabilities afforded by biobanks and big data analytics [89–92].

In each of these cases, data/biospecimens were collected for specific purposes related to health care or health research. The consents provided – or in the case of NBS, the implied consent – did not extend to legal or security uses. Arguably, the bioethical principles and privacy protections governing the original consent were negated when access was allowed after the fact for other purposes. While democratically sanctioned processes or ethical reasoning may have been applied in some cases, as in the use of NBS samples for identification after the 2004 Asian tsunami, in others legal or other considerations were allowed to overrule the protections and understandings in place when the data/biospecimens were provided. These examples illustrate the limitations of the confidentiality protections that researchers are realistically able to offer research participants in many legal environments [93]. Even where legal protections exist (such as Certificates of Confidentiality in the U.S.), their practical efficacy in preventing access has yet to be tested in court, especially when “national security” or other perceived pressing national interests are implicated [94].

### Health resource allocations

A major challenge in presenting an analysis of *unintended* and *unforeseen* consequences is that we cannot know the ways in which particular societal interests develop and new ones emerge. But it is precisely these unforeseen uses we would like to highlight. The following example about health resource allocation is intended to illustrate a possible trajectory of societal interests in which health research data collections might come to find very different uses.

All health care systems currently use some method for rationing the provision of resources [95] and current forms of health care delivery are unlikely to be sustainable in many jurisdictions [96]. In addition to demands associated with a demographic shift in age profile and an ever expanding range of new treatment options, costs of treatment are growing, in some cases dramatically. Drugs to treat rare diseases, which in aggregate may include as many as 1 in 12 Canadians [97] can exceed $300,000 per patient per year, in contrast to an average cost of under $1000 per person per year across the Canadian population [98]. Similarly, cancer drugs may cost tens of thousands of dollars for short term life extension [99]. These pressures on health care systems mean that governments and health insurers have to make increasingly difficult decisions about how to ration limited health resources.

Understandably, attempts toward rationing are controversial, especially when these are seen to be discriminatory. This is evident, for example, in recent media coverage in the UK about non-research uses of identifiable personal health information for risk stratification purposes (see, for example, [100]). Similar programs in the U.S. seek to identify frequent uses of ER admissions for the specific reason of targeting them for additional services [101–104].

Given pressures on health care systems, more drastic measures might be taken in the future to inform and enact rationing decisions. These trends might therefore lead to attempts to access health data collections for purposes of facilitating health resource rationing decisions. Governments and health insurers already have access to large amounts of personal health information about individual citizens, and in some cases legal protections are in place to guard against discriminatory practices. But legal protections are limited, and the types of health data collections available for research (outlined above) arguably extend the information otherwise available to health providers.

Lifestyle factors and personal health information are already used to make decisions by insurers about premiums and, in some instances, to exclude certain people from receiving coverage, as is done with life insurance coverage [105]. In the U.S., the Genetic Information Nondiscrimination Act (GINA), in place since 2009, makes it illegal for health insurance companies or employers (through which people usually access health insurance) to discriminate based on genetic data [106] and the Affordable Care Act enacted in 2010 protects consumers against health insurance discrimination based on pre-existing conditions and against rate hikes based on medical diagnoses [107]. However, neither Act addresses life insurance [105]. In Europe, regulations prohibiting insurance discrimination based on genetic data have been in place for more than two decades, starting in 1990 in Belgium. However, these laws may in some cases be too narrow to fully protect people from the use of data associated with genetic information [108]. Some have also argued that there are benefits to using genetic test results in assessing insurance premiums because doing so allows insurers to be more specific about who is at risk, rather than penalizing everyone in a particular category (e.g., women who have a family history of breast cancer but themselves do not have the particular mutation that would place them at increased risk) [109].

Information about individuals’ health behaviours is arguably of interest in making health resource decisions because behavioural data (smoking, drinking, diet, exercise) are strong predictors of health outcomes [110]. Indeed, incentive-based health programs already rely on such data to shape individuals’ health behaviours, a practice which has been heavily criticized on ethical grounds and has potential for discrimination [111–113]. This potential for discrimination has been well documented among smokers. In contrast to other areas of health and social policy which have worked to counteract stigmatisation of affected groups and individuals, policy on tobacco control in some jurisdictions has actively sought to create stigma against smokers. These strategic stigmatisation efforts are associated with some alarming consequences, including surgeons refusing to treat smokers or pushing them down waiting lists, and family doctors not taking on smokers in their clinics or providing them with lower quality care [114]. Insurance discrimination has also been documented based on risky behaviours like extreme sports (sky diving, etc.) [115].

Risk stratification can be used to ensure effective allocation of limited health care resources across a population. Further, individuals whose behavior puts them at increased health risk might legitimately be required to accept the financial burdens of those risks. However, criteria for determining ‘effective allocation’ and classifying what kinds of behaviour qualify as ‘high risk’ inevitably require value based judgments. Therefore, all decisions relating to health care coverage are inevitably political [116] with strong moral dimensions [117]. We are here not advocating for or against particular approaches to health care allocation (e.g., luck egalitarianism). Rather, our point is that the use of health data to help inform health resource allocation is not simply a technical step in refining such decisions; the way they are used depends on particular values which should be subject to democratic scrutiny. Moreover, it is typically only socially privileged individuals who are able to dedicate their lives to health pursuits in ways that conform to merit based norms. With increasing pressure on financial sustainability of health care systems, it is not difficult to envisage increasing pressure to adopt merit based allocation of health resources, with those who are more “responsible” with their health and conform to rationalistic norms of health decision-making and personal life style choices rewarded with greater access to health resources, while non-conformers (e.g., smokers; vaccination refusers; screening non-attenders; non-compliant patients; sedentary habits) are punished with diminished access [118]. Social media data are a prime candidate to support such assessments. Indeed, some insurers currently offer incentives to people who provide their health information (particularly behavioural data) in exchange for a discount on premiums, as long as they show healthy behaviour [119–121]. This places individuals who conscientiously object to this practice at a disadvantage to the extent that the discounts offered to those who comply are likely offset by increased premiums for those who refuse.

### Human rights abuses and eugenics

Some of the health data collections (fitness trackers, social media, DTC genetic testing) we consider here have been developed relatively recently. However, population data have been collected historically and the examination of how certain types of data collections have been (mis)used for eugenic and similar purposes offers important insights. Seltzer and Anderson [122] describe ten historic cases in which documentary evidence exists to link the use of population data systems with human rights abuses. In some of these cases the data systems were “neutral” or even intended for population benefit, before being subverted for eugenic purposes. For example, in the Netherlands a population registration system was established in part to conduct social research, which was subsequently adapted in 1941 for the apprehension of Dutch Jews who were then deported to death camps. The death rate among Dutch Jews (73 %) was dramatically higher than that among Jews in France (25 %) and Belgium (40 %), as well as Jewish refugees living in the Netherlands during the Nazi occupation. Seltzer and Anderson argue that this was largely due to the fact that the registration system in the Netherlands facilitated the apprehension of Dutch Jews. Critically, the point here is not that the collection of a particular form of data resulted in human rights abuses, but that their availability facilitated such abuses.

Consideration of the ELS implications of health data has predominantly occurred in the context of developed countries, characterized by stable democracies that pride themselves on good human rights records (irrespective of evidence to the contrary). As a consequence, the macro-political context within which ELS issues are debated is often presumed to be constant. Seltzer and Anderson’s analysis highlights the flaws in this assumption. Ideological and other shifts in national sentiment can dramatically alter circumstances for particular groups, threatening their status as citizens and personal security. Given the potentially extreme longevity of electronic data collections, significant shifts in macro-political environments need to be considered not only a possibility but a certainty. Recent increases in immigration to Europe, for instance, are associated with an upsurge in support for rightwing political parties. Given that many of these parties across Europe explicitly target immigrants or specific minority groups, it is not unreasonable to suppose that if they gained political power, they might attempt to gain access to any population data systems allowing them to further discriminatory goals. Similarly, at least one of the leading contenders of the Republican nomination for the 2016 U.S. presidential elections strongly supports the forced deportation of any immigrants who are in the country without legal authorization, barring any refugees from Syria from entering the country [123], and has considered the possibility of keeping a database specifically on Muslims [124].

Our point here is not that we predict that databases currently being constructed will be used for such purposes. Rather, our point is that such uses are conceivable (it has happened before). Therefore, it is an oversight that the ELSI literature has not seriously discussed the potential link between utilization of population and health data by authoritarian or populist governments for ends that violate human rights.

### Data security and other uses of health data

The potential for harmful secondary uses of health data has been recognized by some government agencies. In a 2003 report focusing on the ethical, legal and social implications of developments in genetics, the Australian Law Reform Commission noted controversial uses of genetic information relating to law enforcement, immigration, and many other public domains [125]. Perhaps more concerning, a joint report, *National and Transnational Security Implications of Big Data in the Life Sciences*, by the American Association for the Advancement of Science (AAAS), the Federal Bureau of Investigation (FBI) and the United Nations Interregional Crime and Justice Research Institute (UNICRI) observed that with increasing amounts of health data, more data sharing, and improvements of analytic tools, there is an associated increase in risks for stealing sensitive data and “to inflict harm on individuals or groups, support a criminal enterprise, or disrupt Big Data applications to cause negative economic, political, or other societal outcomes” (p. 17) [126]. The report further concludes that “risks can range from inappropriate access to sensitive data such as the numerous examples of cyber attacks to healthcare databases …, to the use of Big Data analyses in designing harmful biological agents.” (p. 34) [126].

Given extensive efforts to facilitate the sharing of health data across jurisdictional boundaries, such as the Global Alliance [127], attempts to compel access to health data by foreign governments once data have been shared across jurisdictional boundaries must also be considered, and the dynamic nature of political environments in countries beyond the origin of the health data cannot be ignored. Concerns must be acknowledged, for example, of such data being used to support new forms of eugenics in autocratic regimes outside of the jurisdiction within which the data were originally collected. Interestingly, the Court of Justice of the European Union (C-362/14) recently found the U.S. Safe Harbour agreement invalid, in part due to the Snowden revelations about NSA access. The agreement previously formed one of the bases for transfer of data from the EU to the U.S., and data transfer from the EU to the U.S. is currently legally challenging [128]. Health data might also be used for homeland security. Legislation such as the USA Patriot Act suggests at least the possibility of government agencies forcing access to health data repositories for purposes of improved surveillance. Indeed, substantive efforts to link health and homeland security databases have been underway in the U.S. at least since the terrorist attacks of 9/11 [129]. Or, health data might be used to identify individuals with propensities (“genetic predictors”) for criminal activity [130], or vulnerability to addiction [131].

We hope that these examples of secondary uses suffice to make our point. In some instances, they are more hypothetical (e.g., use of health data for border security); in others, precedents for secondary uses have already been established in some jurisdictions with varying public responses (e.g., newborn blood spots used for forensic purposes; biobank samples used to adjudicate in biological kinship disputes). It is also important to note that the health data collections we described above all exist within different legal frameworks, with different levels of electronic security, and hence affording different degrees of legal and illegal access. It might be argued, for example, that genetic information is well protected in government funded research databases, but that the same (or similar) information might not be as well protected legally and electronically when collected and held by direct-to-consumer genetic testing companies. In all cases, we have sought to illustrate that the existence of health data collections creates affordances for uses that are not envisaged in their original purpose.

## Conclusions

The implications of large amounts of health data being collected, stored, and shared for health research cannot be understood in isolation from other societal trends. In particular, new challenges relating to big data are recognized for all kinds of electronic information unrelated to health, such as consumer purchasing behavior, internet browsing patterns, and the vast amounts of personal information available about every one of us on the web. Critical scholarship in these contexts points to the limitations of protecting privacy by giving individuals more control of data about them [132], and of relying on “notice-and-consent” as cornerstones of online privacy [133, 134]. Given the nature of digital data with its potentially indefinite lifespan and unprecedented capacities for re-analysis and information sharing, we need to adopt a much longer time horizon in examinations of societal implications. Indeed, addressing some of the issues we have raised in this paper is likely an important step toward establishing justifiable, legal data sharing solutions for the long term within the health care and research sector. We also need to be broader in the scope of our considerations. Limiting privacy considerations to issues around informed consent and de-identification (or anonymization) tends to focus on implications for the individual. However, information collected in health research clearly has implications for family members and the identifiable ethnic groups to which individuals belong, in the present and the future, particularly when we consider genetic and genomic data. We also need to consider the implications of living in a society in which the collection and electronic storage of health data have become increasingly normalized.

To this end it is worth considering briefly some of the mechanisms we might rely on to protect against misuses of data, and to assess their effectiveness for the kinds of scenarios described above. Computational mechanisms to safeguard data are certainly an important aspect of managing ELS concerns surrounding biobanks and health data, and significant efforts in this regard are taking place [135]. However, as documents such as the *National and Transnational Security Implications of Big Data in the Life Sciences* [126] testify, it would be a mistake to assume that even very sophisticated electronic safeguards cannot be circumvented. Further, computational and technical safeguards in themselves are insufficient in the face of changes in public and political sentiment, especially if these lead to changes in policy or law. Electronic security measures are tools that do not serve to protect against the authorities who are in charge of those tools. In the US, a certificate of confidentiality may be issued on the request of a researcher, if data are deemed highly sensitive; this certificate is intended to permit the researcher to refuse a legal request for identifiable data [136]. Limited case law suggests that certificates of confidentiality can protect against compelled disclosure of data. However, an evolving legal climate in which data are seen as essential to support core governmental functions, such as national security or affordable health care, could result in overriding such certificates. Importantly, many countries do not provide the equivalent of US certificates of confidentiality.

Another mechanism we currently rely on to protect participants in research from harm (unintended or otherwise) are Research Ethics Boards (REBs) or Institutional Review Boards (IRBs). Although they arguably provide many important protections, the scope of IRBs does not protect from the kind of societal consequences we have discussed in this paper. Indeed, consideration of “social consequences” is strictly outside the bounds of U.S. human research protection regulations [137]. In Canada, the TCPS2 does not prohibit REBs from considering societal consequences of research, but nor does it specifically ask boards to consider these. The Oviedo Convention [138] and the Norwegian Act on Medical and Health Research [139] similarly state that the risks and benefits of research must be assessed for the individual, but REBs are not obliged to consider broader societal aspects of the research. And while IRBs have authority over individual research studies, they generally do not have the power to deny a government access to certain forms of data. They certainly do not have the power to restrict uses of data that have been shared beyond their jurisdictional boundaries (note, however, discussion around the need to give greater recognition to group harms of research) [140]. Notably, IRBs also differ in how they handle risk and protect research participants. The literature shows variation between how boards interpret what is considered research and what type of ethics review is necessary [141, 142]. Even if IRBs were to consider some of the broader consequences of using secondary data for one study, this consideration might differ from IRB to IRB. But more importantly, the use of anonymous data for secondary use may also escape research ethics review altogether under current (U.S. and other) regulations. And if research is done in private institutions, it too may not be reviewed by IRBs. As such, IRBs might offer little to no protections to individuals in addition to insufficient consideration of social consequences.

Anonymisation of data by itself is also not an entirely satisfactory or feasible solution to protect data [143–145] nor does it meet the needs of those who would rather not have their data included in particular types of research [146]. Further, in some cases, researchers may need identifiable or re-identifiable data [147]. For example, some of the health data sources we have described are by their nature associated with personal identifiers (e.g., clinical health data; social networking profile). Further, when it comes to collecting data for health research, the more the data is stripped of information that could be used to identify an individual, the less the data are useful to answer certain types of research questions [148]. There is therefore an incentive and societal value to the collection of identifiable data. Finally, anonymisation can often no longer be guaranteed because in many instances it is possible to re-identify individuals from “anonymized” health data [149–151]. In some instances, such as the Personal Genome Project, the idea that personal information could be private in the modern digital world is rejected as unfeasible. Rather than attempting to protect the privacy of research participants, the project leaders thus focused on recruiting participants willing to accept the risks associated with having their genomes published publicly (though we would argue that the conceptualization of such risks was likely not sufficiently broad). Finally, even participant-centric consent models [152], while undeniably an important step forward in resolving challenges relating to informed consent in large scale research platforms, in themselves do not guard against the type of problems we have outlined in this paper. Indeed, most of the mechanisms we typically rely on for human subjects protection in research do not address issues of access over which the host of the data platform has no control (e.g., IRBs do not have the authority to resist government mandated access). It is also of concern that the recent report on *National and Transnational Security Implications of Big Data in the Life Sciences* cited above further notes that “Beyond access controls, encryption, and other common data and cyber security technologies, no solutions exist that prevent or mitigate attacks on databases or the cyber infrastructure that support Big Data in the life sciences, which could result in consequences to the life science, commercial, and health sectors.” (p. 17) [126].

If concerns about unintended use of health research data are to be taken seriously, and if current safeguards are indeed insufficient, what might be some appropriate steps to take in guarding against particular forms of unintended consequences? At this point, we have no concrete solutions to offer. Our aim in this paper has been to raise issues, which we believe have been neglected in existing ELSI discussions on the topic. Our hope is that this paper will generate more vigorous debate of these issues among ELSI and other scholars.

Along with others we believe that a key consideration lies in fostering greater public discourse and transparency about these issues [153–155] and in the development of strong and independent governance structures for biomedical data and sample repositories [32, 156, 157]. However, to date little effort has gone toward articulating the particularities of how such governance might look like. What efforts have gone towards articulating governance structures for biobanks point towards greater public participation and control of biomedical data and samples at an institutional level [158, 159]. Winickoff’s proposal for the use of charitable trust models to structure biobanks and their relationship with tissue donors provides excellent guidance in this regard, with increased emphasis on responsible stewardship on the part of the biobank, and increased possibilities for meaningful involvement in governance on the part of donors [160]. However, even these models do not protect from many of the issues we discussed here, nor are they implementable for all forms of data collections.

To address the problem of the unsanctioned secondary use of health data at a governmental level, health data governance needs to have deep democratic roots. These governance structures need to be sufficiently strong and independent to be able to withstand governmental and other equally strong pressures to redirect use of data resources away from the originally intended purposes without legitimate democratic scrutiny. With regard to transparency and public discourse, recent efforts to include a voice in biobank policy via deliberative public engagements are a good start [161], and similar methodologies could be developed for other data collections, as has been proposed for implementing predictive analytics in healthcare [70]. Community-led groups constructing health research platforms also provide important precedents with regard to alternative models for governing health research and data [162]. However, while these efforts do create mechanisms for the direct involvement of lay publics, patients, and research participants in the governance of health data collections, they have so far been limited in the extent to which they elevate debate of the issues to truly national levels. Moreover, deliberative and participatory mechanisms that help inform the governance of health data collections do not in themselves provide protection against government or court ordered access.

Secondary uses of health data are not necessarily undesirable. For instance, their use in helping to identify victims in mass casualty events, while certainly not unanimously acceptable, is likely to find strong societal support (see also [163], for other beneficial secondary uses of data and [70] for suggestions on minimizing potential harms). In other cases, such as the hypothetical example of using health data collections to inform health resource allocation, the degree of societal support would likely change over time and depend on contextual factors such as dominant ideological commitments and economic conditions. Support for singling out high users of health care, for instance, may vary depending on whether the underlying purpose is deemed discriminatory or supportive. Yet other potential uses, such as those relating to human rights abuses, should be guarded against no matter what the dominant societal commitments are at any given time in any given place. No matter that the probability of such abuses might be assessed as being very small, the outcomes should they occur would be catastrophic (as illustrated in historical examples). Frustratingly, it is these types of unforeseen and unintended abuses of health data that will likely be the most difficult to guard against. Many of the examples of adverse uses we have discussed in this paper might be guarded against with adequate legal protections within jurisdictions and strong binding data sharing and custodianship agreements between jurisdictions. However, these protections will not guard against strong ideological shifts resulting in changes in government. Developing institutional, legal, and other safeguards to protect against possible encroachment of future governments seems daunting to say the least. And perhaps it is not possible. But at minimum, discourse about these issues needs to occur on political levels at which meaningful democratic guidance can be deliberated. These kinds of activities are not yet sufficient to address fully the concerns we have articulated here, but they point in the right direction. In all likelihood, effective ways to safeguard against (adverse) unintended and unforeseen uses of health data collections will require at a minimum: a) a combination of legal frameworks based on broad and inclusive deliberations on a societal level, b) technical infrastructure in line with these frameworks, which would make it difficult for changing governments to significantly amend the purpose(s) of the health databases, and c) policy at the level of biobanks and other health data platforms, which raises awareness of the possibility of encroachment on the resource for non-health research purposes, as well as providing more fine-grained dynamic control in regulating details about data usage. We realize that this is a no small task, but are of the opinion that now is the time to consider debating the need for such safeguards.

## Abbreviations

CDC: Centers for disease control and prevention (U.S.A.)
CIHI: Canadian Institute for Health Information
dbGaP: Database of genotypes and phenotypes (U.S.A.)
DTC: Direct-to-consumer
ELSI: Ethical, legal, social implications
EMR: Electronic medical record
IRB: Institutional review board
NBS: Newborn screening
NHS: National Health Service (U.K.)
NIH: National Institutes of Health (U.S.A.)
NSA: National Security Agency (U.S.A.)
REB: Research ethics board
